# Field Application of Nanoscale Polymer Microspheres for In-Depth Profile Control in the Ultralow Permeability Oil Reservoir

**DOI:** 10.3389/fchem.2020.00805

**Published:** 2020-10-14

**Authors:** Ganggang Hou, Wenyue Zhao, Yuqin Jia, Xinyu Yuan, Jian Zhou, Tongjing Liu, Jirui Hou

**Affiliations:** ^1^Unconventional Petroleum Research Institute, China University of Petroleum, Beijing, China; ^2^Oil & Gas Technology Research Institute of Changqing Oilfield Company, Xi'an, China; ^3^BeiJing Jinshi Liyuan Science Co., Ltd., Beijing, China

**Keywords:** nanoscale polymer microspheres, ultralow permeability oil reservoir, practical application, performance evaluation, in-depth profile control

## Abstract

Much research has been carried out on nanoscale polymer microspheres (PMs) in laboratories in recent years. However, there are limited reports on the practical application of nanoscale PMs in ultralow permeability reservoirs. This paper reports a field application case of nanoscale PMs for in-depth profile control in the ultralow permeability oil reservoir. In the paper, the characteristics of the reservoir and the problems faced during development are analyzed in detail. Then, the PMs with calibration diameters of 300 nm and 800 nm are researched by evaluation experiments, and are selected for in-depth profile control in the ultralow permeability oil reservoir. Finally, according to the effect of the pilot application, the performance of PMs is evaluated, and a more suitable size for the pilot test reservoir is determined. The experiment's results show that the PMs have a good capacity for swelling and plugging. For the PMs with a calibration diameter of 300 nm, the final equilibrium swelling ratio is 56.2 nm·nm^−1^, and the maximum resistance coefficient and the blocking rate after swelling are 3.7 and 70.31%, respectively. For the PMs with a calibration diameter of 800 nm, the final equilibrium swelling ratio is 49.4 nm·nm^−1^, and the maximum resistance coefficient the blocking rate after swelling are 3.5 and 71.42%, respectively. The performance evaluation results show that nanoscale PMs can be used for in-depth profile control in the ultralow permeability oil reservoir. After the application of PMs in the pilot test area, the average water cut decreased by 10.4%, the average liquid production of single well-increased by 0.9 t/d, and the average thickness of the water-absorbing layer increased by 1.77 m. Comparing the dynamic data variation of well-groups using the PMs with the calibration diameter as 800 nm and the calibration diameter as 300 nm, it indicates that, for the pilot test area, PMs with a calibration diameter of 300 nm are more suitable than PMs with a calibration diameter of 800 nm.

## Introduction

The demand for petroleum energy plays a vital role in global energy supply (Asif and Muneer, 2007). Water-flooding is the most common development method in the primary or secondary oilfield development, because of its convenient application and low cost (Afeez et al., 2018). However, with the development of water-flooding, the heterogeneity of reservoirs will become more and more serious (Sang et al., 2014). In the later stage of oilfield development, the difficulty of water-flooding is increasing, and the efficiency of water-flooding is decreasing (Ji et al., 2017). This phenomenon results in lots of oil remaining in the reservoir, which cannot be produced by conventional development methods (Pu et al., 2016; AfzaliTabar et al., 2017). To combat this, enhanced oil recovery (EOR) technology can be used to develop this oil. However, there are so many EOR methods, such as chemical flooding and gas flooding, and which method should be used in the oilfield, depends on the characteristics and main problems of the reservoir.

Low or ultralow permeability oil reservoirs are mainly characterized by their low porosity, low permeability, small radius of pore-throat, and widespread distribution of natural fractures (Lin et al., 2015; Liu et al., 2018). The characteristics of low permeability reservoirs makes it necessary to use artificial fracturing technology during the development of the reservoir. The artificial fractures formed by artificial fracturing can connect with natural fractures, thus the development efficiency of low permeability oil reservoirs will significantly increase (Fletcher et al., 1992; Diwu et al., 2018). However, the permeability of these fractures will gradually become higher after the long-term injection of water, and that will improve the heterogeneity of low permeability oil reservoirs (Zhao et al., 2018). This in turn leads to low permeability oil reservoirs facing many problems, such as rapid breakthrough of water, non-uniform water-absorbing of injection wells, and low water flooding efficiency (Liu et al., 2018). Profile control technology is considered to be an effective method to solve such problems (Jia et al., 2018).

Profile control technology can be divided into conventional profile control and in-depth profile control, according to the plugging positions (Dai et al., 2010). The conventional profile control method mainly plugs the high-permeability channels near the well, while the in-depth profile control method mainly plugs the high-permeability channels in the deep part of the reservoir (Zhao et al., 2014). For the ultralow permeability oil reservoir, in-depth profile control is more suitable than the conventional profile control. This is because in the ultralow permeability oil reservoir, fractures including the artificial fractures and natural fractures are distributed widely. After the near well is plugged, the subsequent injection water will flow around these areas and then return to the high-permeability channels (Zhou et al., 2017). A key component of the in-depth profile control technology of ultralow permeability oil reservoirs is to ensure that the agent can reach the deep part of the reservoir and effectively plug the high-permeability channels (Zhang and Zhou, 2008; Jia et al., 2019). However, this is impossible for most conventional profile control agents, as they usually cannot flow through the small pores and throat (Li et al., 1993; Tu and Wang, 2011). The nanoscale polymer microspheres (PMs) have attracted more and more attention as in-depth profile control agents in recent years (Wu et al., 2018).

PMs are a viscoelastic plugging agent with a 3-D structure that can absorb much more water as compared to their own mass and that make it hard to release the absorbed liquids even under high pressure (Yang et al., 2017). However, these characteristics are not enough for practical application in oilfields. They also need to have other excellent properties, such as stability and rheology. Many researchers have done a lot of research on these properties (Kawaguchi, 2000; Kang et al., 2015; Lin et al., 2015; Nandwani et al., 2020). The research results show that the stability of the PMs plays a decisive role in deep profile control (Afeez et al., 2018). If the stability is not good, the PMs will be absorbed in near the well-bore and plug the injection channel near the well-bore; thus, the purpose of in-deep profile control will not be achieved (Dai et al., 2017). In order to obtain PMs with better stability, many types of PMs have been developed (Hua et al., 2014; Mehta et al., 2015; Yang et al., 2017b). All these polymer microsphere systems have shown excellent characteristics in laboratory evaluation studies. However, there are limited reports about their application effect in oilfields, especially in ultra-low permeability reservoirs.

This paper reports on a field application case of nanoscale PMs for in-depth profile control in ultralow permeability oil reservoirs. In the paper, the characteristics and the problems of Block A in the Changqing oilfield are analyzed in detail. Then, the PMs with calibration diameters of 300 nm and 800 nm are selected based on the radius distribution of the pore-throat of Block A. For ensuring the swelling and plugging properties of PMs, a hydration swelling experiment and plugging evaluation experiment have been done. Based on the experimental evaluation results, the nanoscale PMs are used in the pilot test area for in-depth profile control. Finally, according to the variation of dynamic data of production wells and injection wells, the performance of PMs in the pilot test area are evaluated.

## Reservoir Background

### Field and Reservoir Description

**Figure 1:**
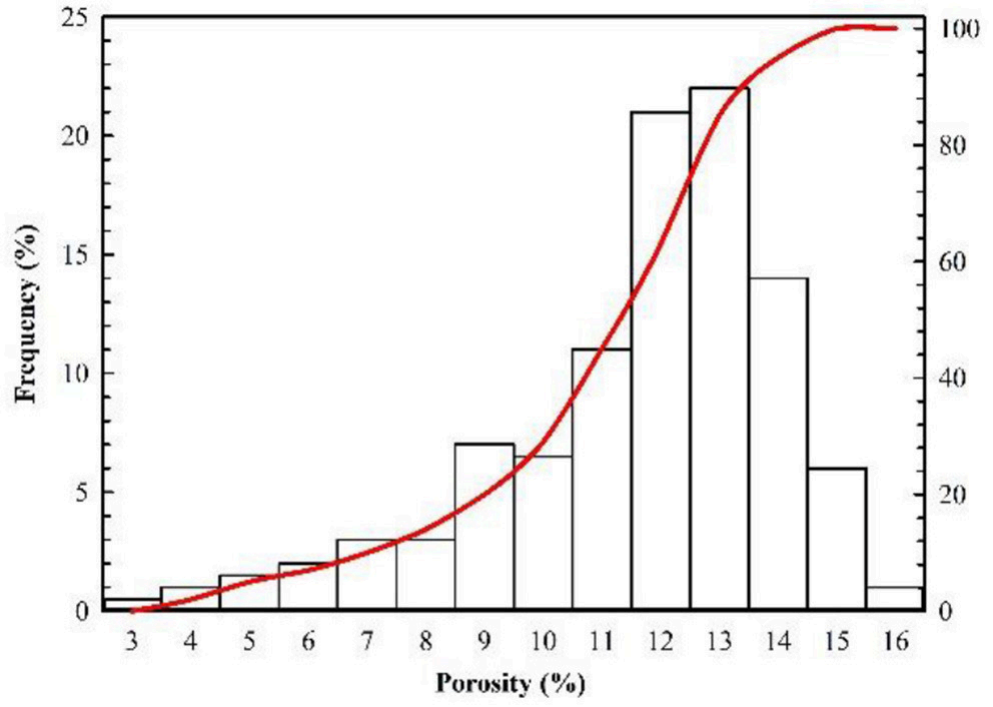
Distribution histograms of porosity.

**Figure 2:**
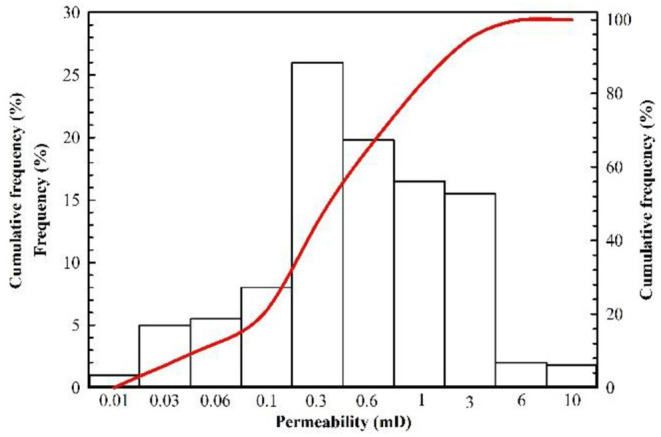
Distribution histograms of permeability.

**Figure 3:**
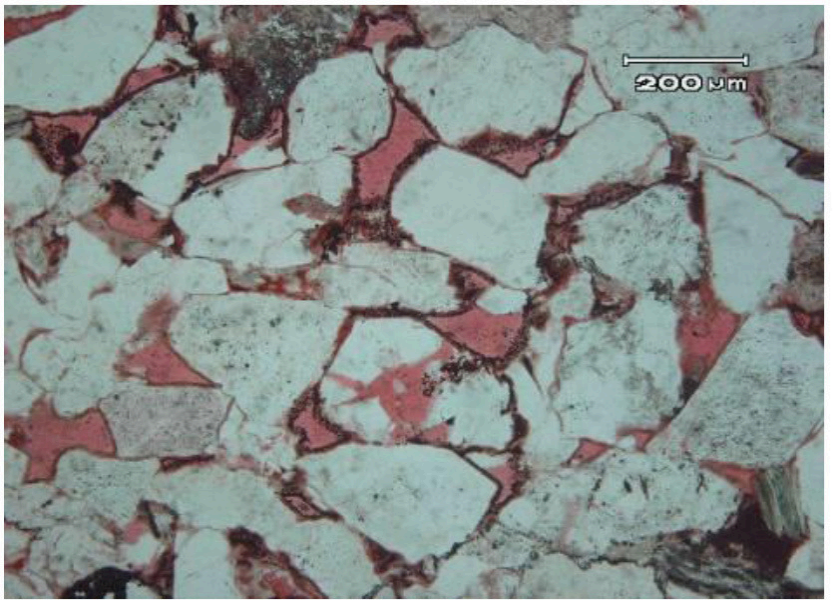
Core casting thin sections sample.

**Figure 4:**
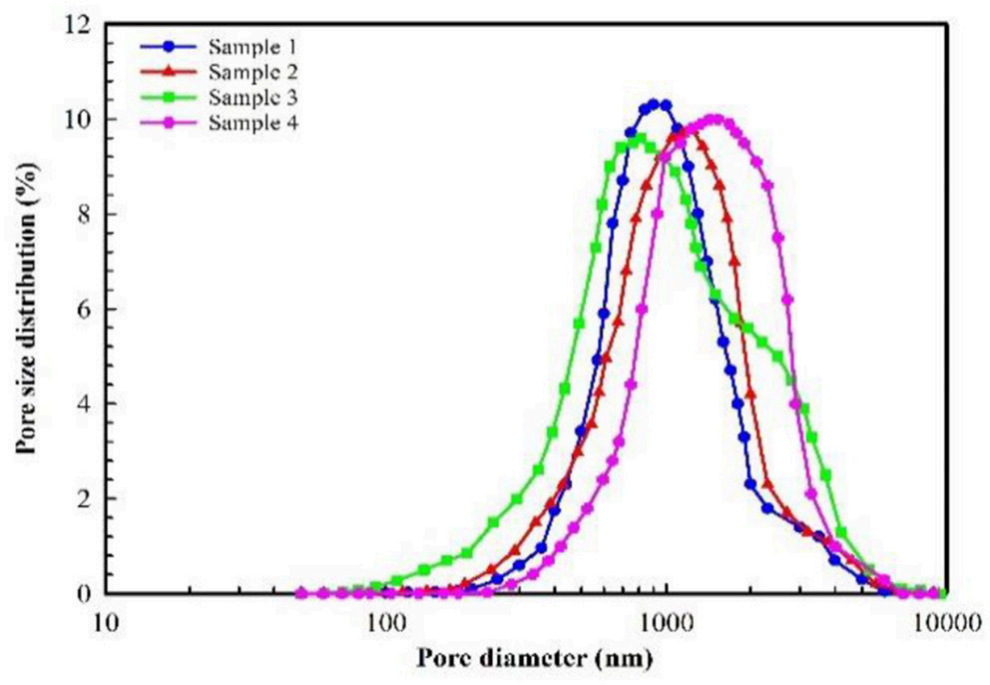
Radius distribution curve of pore-throat.

The Changqing Oilfield is composed of many oil-producing reservoirs, such as Block A, which is represented by its ultralow permeability. The main production layer of Block A is the Chang 8_2_, and its depth ranges between 2,480 and 2,580 m. The thickness of the oil layer varies with net to gross ratio, but the average thickness of the oil layer is 13.69 m. The porosity and permeability of Block A are obtained from the coring results of 18 wells, and their distribution histograms are shown in Figures 1, 2. The porosity mainly ranges between 5 and 15%, and its average value is 10.02%. The permeability mainly ranges between 0.03 and 3 mD, and its average value is 0.68 mD. The results of casting thin sections indicate that the pore types of Block A are mainly intergranular pores, as shown in Figure 3. Based on the analysis of mercury injection experimental data, the radius distribution of the pore-throat for Block A mainly ranges between 500 and 7,000 nm. The radius distribution curves of the pore-throat are shown in Figure 4. The initial pressure and temperature of Block A are 17.5 MPa and 70.4°C, respectively. The fluid properties under reservoir conditions are shown in Table 1.

**Table 1:** Fluid properties under reservoir conditions.

**Fluid properties**	**Value**
Oil density	0.733 g/cm^3^
Oil viscosity	1.403 mPa·s
Oil volume factor	1.297
Oil freezing point	19°C
Water salinity	43,000 mg/l
Water type	CaCl_2_

Primary oil production started in 2008 for Block A, but water injection was initiated in 2007 to maintain reservoir pressure. In the early stage of production, the average daily oil production of a single well was 5.92 t/d, and the average water cut of a single well was 16.3%. The production dynamic data indicated that at the beginning all the production wells had very good performances. However, with continuing development, the production performance of this block gradually became worse. As of August 2016, the average daily oil production of a single well-dropped to 1.34 t/d, and the average water cut of a single well-increased to 62.3%. The results of water injection profile tests showed that the characteristic of non-uniform water-absorbing profile was significant, as shown in Figure 6. The development of Block A was facing many problems, such as the rapid rise of water cuts, the rapid decline of oil production, and the poor performance of water- flooding. The application of PMs for in-depth profile control is considered as an effective method to solve these problems.

### Pilot Test Area Description

**Figure 5:**
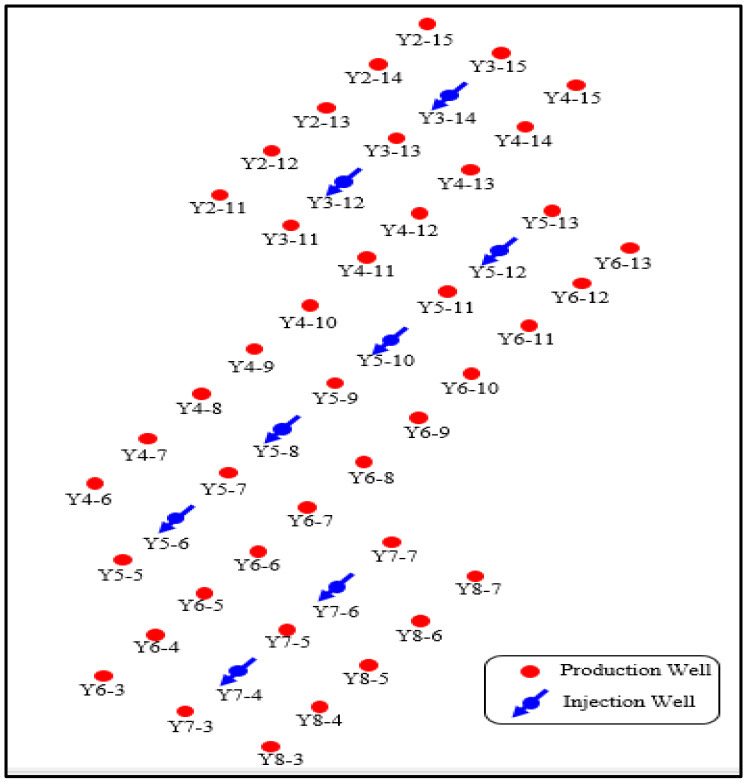
Well-location map for pilot test area.

**Figure 6:**
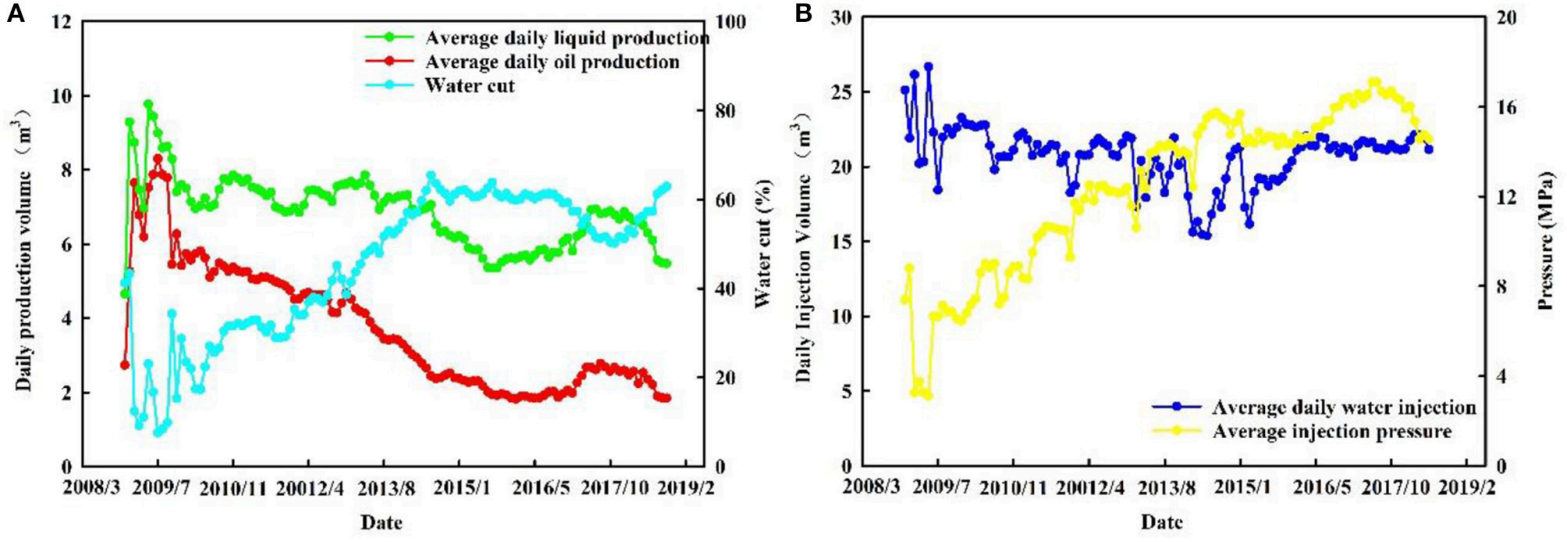
Dynamic data: **(A)** production dynamic date of oil wells; **(B)** injection dynamic date of water wells.

In order to evaluate the performance of PMs in-depth profile control in the field application, in September 2016, Changqing Oilfield selected eight well-groups, including eight injection wells and 42 production wells, in Block A for a pilot test. The well-location map and the dynamic data are shown in Figures 5, 6. As of August 2016, in the pilot test the average daily liquid production of a single well was 5.8 t/d, the average daily oil production of a single well was 1.9 t/d, and the average water cut of a single well was 60.6%. The non-uniformity coefficient of liquid production and water cut was 0.66 and 0.45, respectively. For the injection wells, the average daily injection rate of a single well was 21.4 t/d and the average injection pressure of a single well was 14.6 MPa. The results of a water injection profile test for five injection wells are shown in Figure 13, and they indicate that for the pilot test area the characteristic of non-uniform water absorption profile was significant.

Selecting the appropriate particle size for PMs is the key to the successful application of PMs for in-depth profile control (Li et al., 1993; Hua et al., 2013; Wu et al., 2018). If the particle size of PMs is too small, they will directly flow to the production well with the injected water (Sun et al., 2006). If the particle size of PMs is too large, they will not reach the deep part of the reservoir (Liang et al., 2016). At present, the main method for selecting the particle size of PMs is based on the three-ball bridging theory proposed by Abrans. This theory demonstrates that the plugging rules of suspended solid particles at the pore and throat are as follows: (1) when the particle size is larger than 1/7 of the pore-throat diameter, the suspended solid particles can completely plug the pore and throat; (2) when the particle size is 1/3 to 1/7 of the pore-throat diameter, the suspended solid particles can partially enter the reservoir and slightly plug the pore and throat; (3) when the particle size is < 1/7 of the pore-throat diameter, the suspended solid particles can completely enter the reservoir and cannot plug the pore and throat. Therefore, the plugging effect is best when the particle size of the PMs is equal or slightly larger than 1/3 of the pore-throat diameter (Wang et al., 2006; Zeng et al., 2012). In order to achieve the purpose of in-depth profile control, the particle size of PMs cannot be too large, otherwise they cannot enter the deep part of the reservoir. For the pilot test area, the radius distribution of pore- throat mainly ranges between 500 and 7,000 nm. 1/3 of the pore-throat diameter is between 333 and 4,667 nm. Therefore, considering the hydration swelling property, the PMs with a calibration diameter of 300 nm and 800 nm were selected for in-depth profile control.

## Experimental Section

### Materials

The calibration diameter of PMs used in the experiment were 800 nm and 300 nm. The main ingredient of PMs is polyacrylamide, which was provided by Changqing Chemical Group Co. The anhydrous ethanol (CH_3_CH_2_OH, purity above 99.5%) was used as a dispersant for measuring the diameter of PMs before swelling. The composition of the simulated formation water used in this study is shown in Table 2. The natural cores used in this study were obtained from Chang 8 layer. The properties are shown in Table 3.

**Table 2:** Ionic composition of formation water and simulated formation water.

**Ion**		**Na^**+**^/K^**+**^**	**Ca^**2+**^**	**Mg^**2+**^**	**Cl^**−**^**	**${\text{SO}}_{4}^{2-}$**	**HCO_**3**_^**−**^**	**Total**
Ion content (mg/L)	Simulated formation water	8,105	7,692	31	24,667	14	161	40,670
	Formation water	7,185	8,571	162	2,6679	11	381	42,989

**Table 3:** Core properties.

**Core number**	**Length/cm**	**Diameter/cm**	**Porosity/%**	**Permeability^a^/mD**
1#	10.005	2.515	15.31	15.9
2#	10.005	2.515	15.23	16.1
3#	10.005	2.515	14.24	16.4
4#	10.005	2.515	15.19	16.0

a *Gas log permeability*.

### Experimental Setup

The evaluation experiments of PMs were divided into two parts. One was the experiment of particle size measurement, the other was the displacement experiment.

The experiments of particle size measurement were mainly to evaluate the hydration swelling properties of PMs. The main equipment used in these experiments were Ultrasonic Instrument (produced by Tianjin Autoscience Instrument Co., Ltd.) and Nanoparticle Size Analyzer (produced by Beckman Coulter, USA). Ultrasonic Instrument was primarily used to disperse the PMs solution; its ultrasonic frequency and rated power were 40 KHz and 120 W, respectively. Nanoparticle Size Analyzer was mainly used to measure the diameter of PMs; its measure range is 0.6 nm - 7 μm. Other equipment, such as thermostats, quartz cuvettes (10 ml), and electromagnetic stirrers, were also used in these experiments.

**Figure 7:**
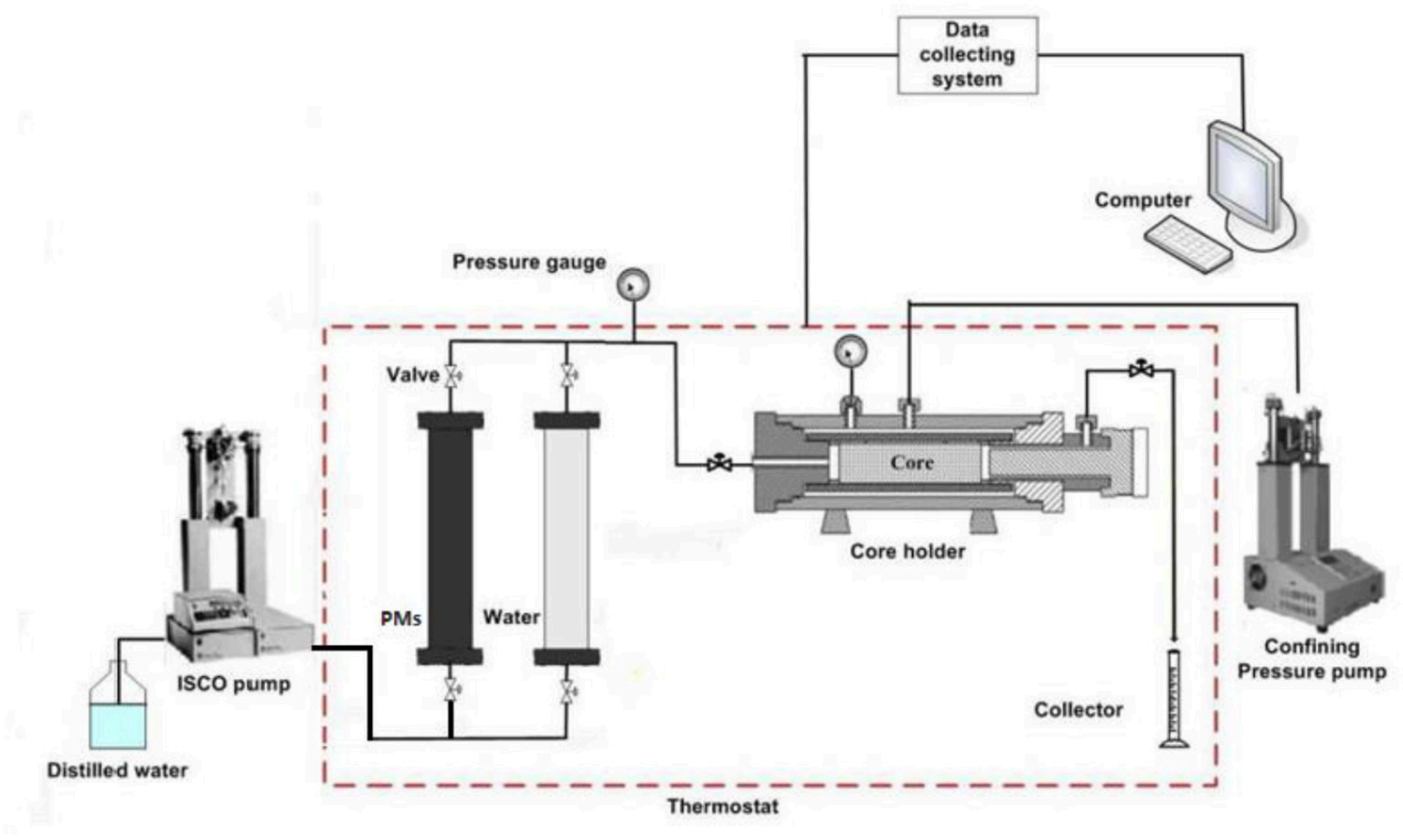
Schematic of the experimental setup.

The displacement experiment was mainly used to evaluate the plugging properties of PMs. The main equipment used in these experiments were the displacement device and the production fluid detecting device. The experimental setup consisted of a syringe pump, core holder, two cylinders, a constant temperature system, and a pressure transducer connected to a desktop computer for continuous recording of the inlet pressure. The schematic of the experimental setup is shown in Figure 7. The syringe pump (Model 260D, Teledyne ISCO, Lincoln, NE) was used to inject fluids at a desired flow rate. Its maximum pressure is 30 MPa, and the flow rate ranges from 0.01 to 20 ml/min.

### Experimental Procedure

#### Hydration Swelling Experiment

Nanoparticle Size Analyzer was used to analyze the particle size before and after swelling. The initial particle size was measured in anhydrous ethanol solution at room temperature, while the swollen particle size was measured in the simulated formation water at 70°C. In the particle size distribution curves, *D*_50_ was used to express the average particle size of the microspheres before and after swelling. *D*_50_ is the particle size from the cumulative distribution curve for a probability of 50%. The experimental procedures of hydration swelling properties are summarized as follows:

(1) Take 100 ml of anhydrous ethanol into a beaker and add an appropriate amount of PMs (including 300 and 800 nm). Stir it to get the test sample. Then, measure the diameter three times at room temperature, and calculate the average value as the initial particle size.
(2) Disperse PMs (including 300 and 800 nm) into the simulated formation water. Put it into the thermostat and record the time as 0 h. Set the temperature of the thermostat to 70°C. After 24 h, shake the PMs (including 300 and 800 nm) solution for 30 min, and measure the diameter three times. Calculate the average value as the particle size after hydration. Measure the diameter continuously until the change of particle size is small.

#### Plugging Evaluation Experiment

In order to evaluate the influence of PMs' swelling capacity on the plugging properties, the plugging experiment of PMs (including 300 and 800 nm) before and after swelling were conducted, respectively. Pressure was recorded to evaluate plugging properties. In this experiment, the injection concentration, injection amount, and injection rate of PMs (including 300 and 800 nm) were 0.3%, 0.3 PV, and 0.3 ml/min, respectively. The specific experimental steps were as follows:

(1) First, weigh the cores after they are dried in the thermostats. Then weigh them again after saturated formation water. Finally, calculate the core porosity.
(2) Set the temperature of the thermostat to 70°C. Inject the simulated formation water into the core 1# at a rate of 0.3 ml/min until the pressure is stable, and then record the pressure. Calculate the permeability of the core.
(3) Inject the PMs (including 300 and 800 nm) before swelling into the core at a rate of 0.3 ml/min, and record the pressure. Stop injecting the PMs (including 300 and 800 nm) when the injection volume reaches 0.3 PV.
(4) Inject the simulated formation water again at the same injection rate and record the pressure. End the experiment when the pressure is stable again.
(5) Change the core, and repeat steps 1–4. However, change the PMs (including 300 and 800 nm) injected in step 3 to the after swelling.

## Results and Discussion

### Hydration Swelling Experiment Results

**Figure 8:**
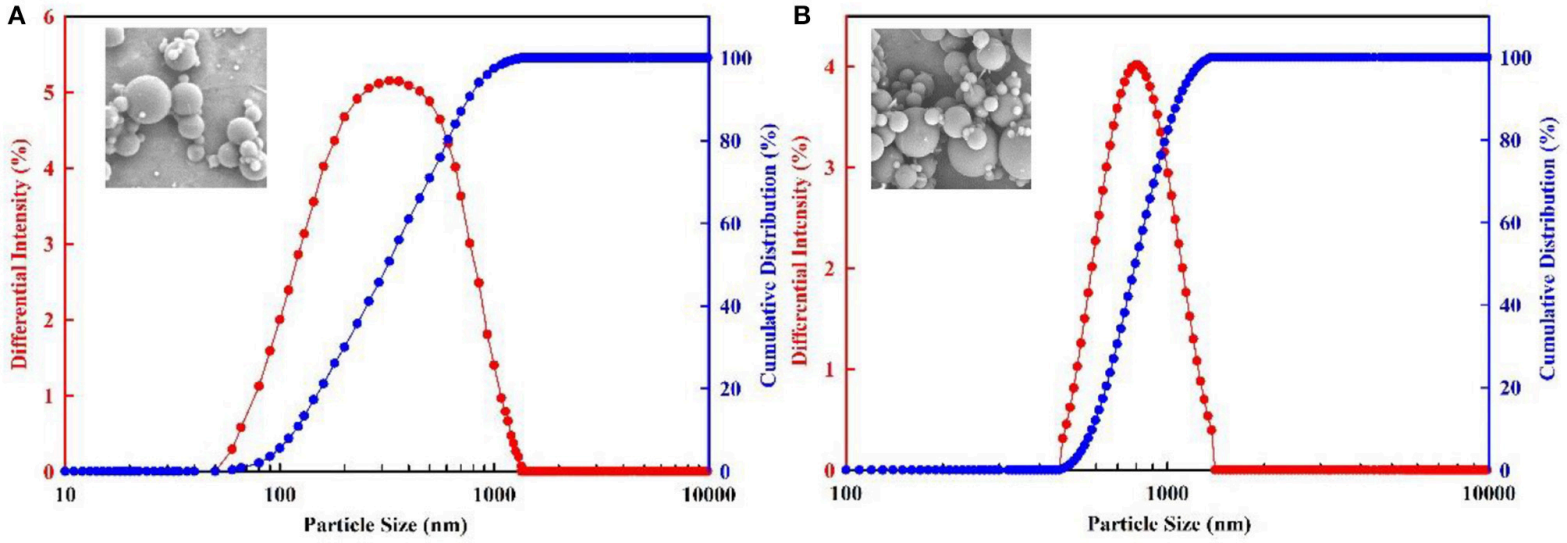
Particle size distribution of PMs before swelling. **(A)** The calibration diameter is 300 nm; **(B)** The calibration diameter is 800 nm.

**Figure 9:**
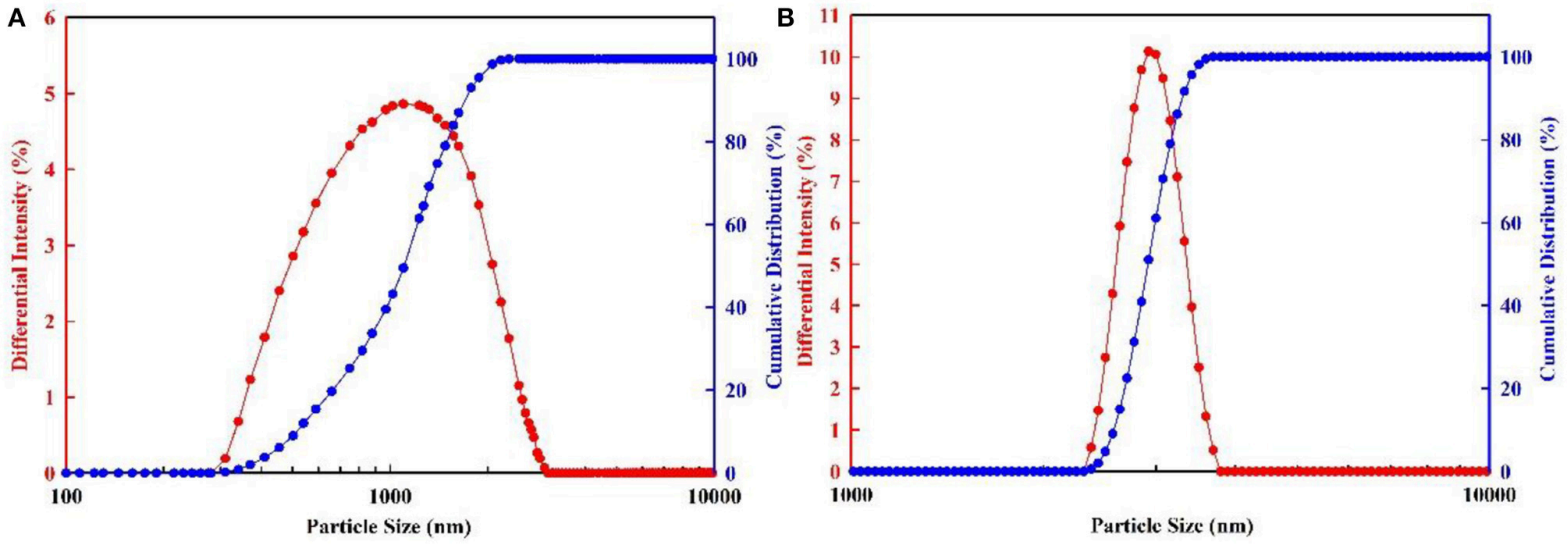
Particle size distribution of PMs after swelling. **(A)** The calibration diameter is 300 nm; **(B)** The calibration diameter is 800 nm.

The particle size of PMs before swelling are measured in the condition of anhydrous ethanol solution and room temperature, as shown in Figure 8, and the particle size of PMs after swelling are measured in the condition of simulated formation water at 70°C, as shown in Figure 9. The three-dimensional (3D) structure of the PMs before swelling can be seen clearly from the environmental scanning electron microscopy (ESEM) image, as shown in Figure 8. As can be seen from the ESEM image, the PMs are spherical particles. The experiment's results show that the particle size of PMs, with calibration diameter as 300 nm and 800 nm, before swelling is 315 and 784 nm, respectively, and after swelling is 1,205 and 2,798 nm, respectively. The PMs can swell many times due to absorbing a lot of water.

The hydration swelling capacity of PMs can be evaluated using the swelling ratio. The swelling ratio was calculated by Eq (1) (Yang et al., 2017a):

(1) $$\begin{array}{r} SR={\left(\frac{D_{t}}{d_{0}}\right)}^{3} \end{array}$$

where *SR* is the swelling ratio of the PMs (in units of *nm*·*nm*^−1^), *d*_0_ is the initial average particle size of the PMs (in units of *nm*), and *D*_*t*_ is the average particle size of the PMs at swelling time *t* (in units of *nm*).

**Figure 10:**
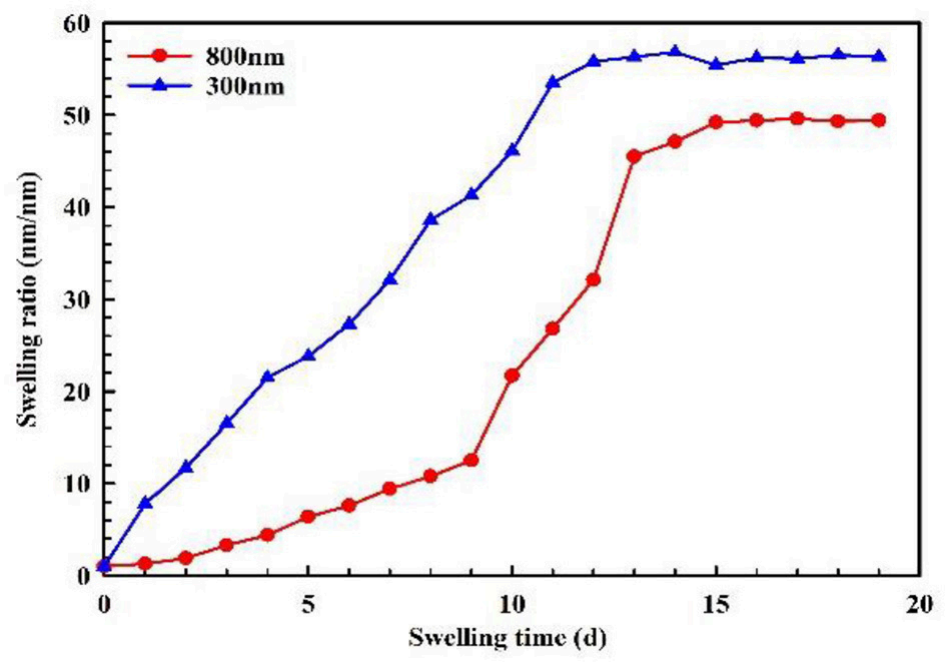
Swelling property of PMs.

The dynamic swelling behavior of PMs are measured every 24 h, as shown in Figure 10. For the PMs with a calibration diameter of 300 nm, initially the rate of swelling capacity sharply increases, and then begins to level off. The final equilibrium swelling ratio is 56.2 nm·nm^−1^, which takes at least 16 days to achieve. For the PMs with a calibration diameter of 800 nm, initially the rate of swelling capacity slowly increases, then begins to sharply increase, and finally tends to level off. The final equilibrium swelling ratio is 49.4 nm·nm^−1^, which takes at least 15 days to achieve. The results of hydration swelling experiments indicate that the PMs, with calibration diameters of 300 and 800 nm, have a good swelling capacity.

### Plugging Evaluation Experiment Results

**Figure 11:**
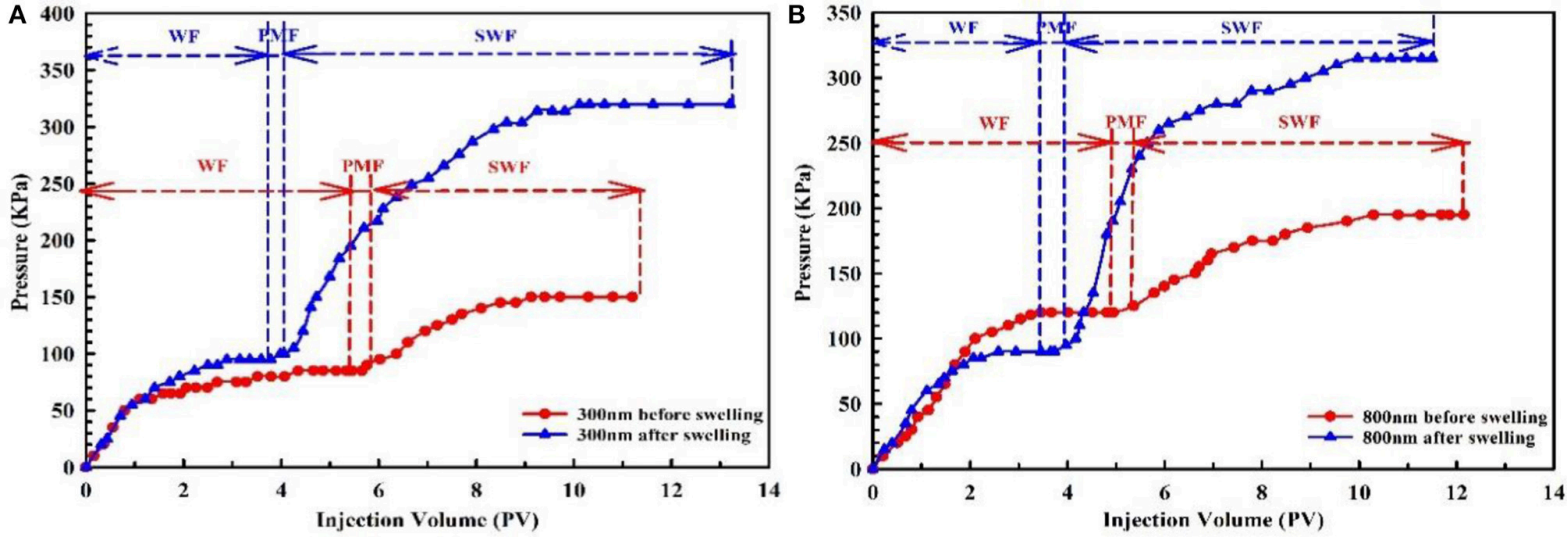
Pressure curve of PMs plugging experiment. **(A)** The calibration diameter is 300 nm; **(B)** The calibration diameter is 800 nm.

The plugging capacity of PMs (including 300 and 800 nm) before and after swelling was evaluated by water-flooding experiments. The PMs with a calibration diameter of 300 nm before and after swelling were injected into cores 1# and 2#. The PMs with a calibration diameter of 800 nm before and after swelling were injected into cores 3# and 4#. Each experiment was divided into three stages: water-flooding (WF), PMs flooding (PMF), and subsequent water-flooding (SWF). At each stage the injection pressure was recorded, as shown in Figure 11. During the displacement experiment, the percolation of the water and PMs conform to Darcy's law. So, the permeability of the core can be calculated by Darcy's law of single phase flow. The resistance coefficient and blocking rate are used to evaluate the plugging capacity of PMs, and this can be calculated using Eq (2) and Eq (3) (Lei and Zheng, 2007):

(2) $$\begin{array}{r} F_{r}=\frac{\lambda_{W}}{\lambda_{P}}=\frac{{\left(K/\mu \right)}_{W}}{{\left(K/\mu \right)}_{P}}=\frac{\Delta P_{P}}{\Delta P_{W}}\times \frac{Q_{W}}{Q_{P}} \end{array}$$

(3) $$\begin{array}{r} \eta =1-\frac{K_{SW}}{K_{W}} \end{array}$$

where *F*_*r*_ and η are the resistance coefficient and blocking rate, respectively. *K*_*W*_, *K*_*P*_, and *K*_*SW*_ represent the core permeability of WF, PMF, and SWF, respectively (in units of μ*m*^2^). μ_*w*_ and μ_*p*_ are the viscosity of water and PMs (in units of *mPa*·*s*). Δ*P*_*W*_ and Δ*P*_*P*_ represent the differential pressure of WF and PMF, respectively (in units of *MPa*). *Q*_*W*_ and *Q*_*P*_ are the injection rate during the process of WF and PMF, respectively (in units of *ml/min*).

The plugging experiment pressure curve of PMs before and after swelling was plotted, as shown in Figure 11. As can be seen from Figure 11A, for the PMs with a calibration diameter of 300 nm before swelling, the stable pressure is 85, 90, and 150 KPa, respectively, at the stages of WF, PMF, and SWF. Meanwhile, for the PMs after swelling the stable pressure is 95, 100, and 320 KPa, respectively, at the same stage. Therefore, it can be calculated that the maximum resistance coefficient of PMs before and after swelling is 1.7 and 3.7, respectively, and the blocking rate of PMs before and after swelling is 43.33 and 70.31%, respectively. As can be seen from Figure 11B, for the PMs with a calibration diameter of 800 nm before swelling, the stable pressure is 120 KPa, 125 KPa, and 195 KPa, respectively, at the stages of WF, PMF, and SWF. Meanwhile, for the PMs after swelling the stable pressure is 90, 95, and 315 KPa, respectively, at the same stage. Therefore, it can be calculated that the maximum resistance coefficient of PMs before and after swelling is 1.6 and 3.5, respectively, and the blocking rate of PMs before and after swelling is 38.46 and 71.42%, respectively. The displacement experiment results show that PMs with calibration diameters of 300 and 800 nm, before and after swelling, have excellent plugging capacity, and the plugging capacity of PMs increases significantly after swelling.

The results of hydration swelling experiments and plugging evaluation experiments indicate that the PMs (including 300 and 800 nm) have two significant characteristics. One is that the PMs can swell many times after absorbing water for a long time, and the other is that the plugging capacity of PMs increases significantly after swelling. These two characteristics are essential for in-depth profile control in ultralow permeability oil reservoirs. For the ultralow permeability oil reservoir, the PMs can enter the reservoir with the injected water, and the particle size will gradually increase during the migration process. When PMs reach the deep part of the reservoir, the particle size reaches its maximum, and at this time it also has a better plugging capacity. Therefore, the high-permeability channels can be plugged, and the purpose of in-depth profile control can be achieved.

## Field Application and Performance Evaluation

### Field Application

The experimental evaluation results indicate that the PMs have a good swelling property and plugging property. Therefore, Changqing oilfield decided to apply the PMs in the pilot test area for in-depth profile control to evaluate its performance in the practical application. There were eight well-groups in the pilot test area, of which four well-groups applied the PMs with a calibration diameter of 300 nm, and the other four well-groups applied the PMs with a calibration diameter of 800 nm. The specific implementation overview is shown in Table 4. For the well groups using the PMs with the calibration diameter of 800 nm, a total of 10 tons of PMs was injected into each injection well from September 2016 to April 2017. The concentration of PMs was the same for every injection well, in which 3 t PMs with a concentration of 5,000 ppm were injected first, and then 7 t PMs with a concentration of 2,000 ppm were injected. For the well-groups using the PMs with a calibration diameter of 300 nm, the injection volume was also 10 tons, but the time was from April 2017 to October 2017. The concentration of PMs was the same, at 5,000 ppm for every injection well. After PMs' injection, the production difference of injection wells and production wells was analyzed to verify the performance of PMs for in-depth profile control.

**Table 4:** Detail parameters of PMs injection.

**Well**	**Injection time**	**Completion time**	**Particle size (nm)**	**Concentration**	**CIV^a^(t)**
Y3-12	2016/9	2017/4	800	5,000 ppm/3t + 2,000 ppm/7t	10
Y3-14	2016/9	2017/4	800	5,000 ppm/3t + 2,000 ppm/7t	10
Y5-10	2016/9	2017/4	800	5,000 ppm/3t + 2,000 ppm/7t	10
Y5-12	2016/9	2017/4	800	5,000 ppm/3t + 2,000 ppm/7t	10
Y5-6	2017/4	2017/10	300	5,000 ppm	10
Y5-8	2017/4	2017/10	300	5,000 ppm	10
Y7-4	2017/4	2017/10	300	5,000 ppm	10
Y7-6	2017/4	2017/10	300	5,000 ppm	10

a *Cumulative injection volume*.

### Performance Evaluation

#### Injection Well Performance Evaluation

After PMs' injection, they will flow into the deep part of the reservoir with the injection water to plug high permeability channels. If the PMs effectively plug high permeability channels, both the injection pressure and the thickness of the water-absorbing layer will increase under the same work conditions. Specifically for the injection pressure, after the high permeability channel is plugged, the flow resistance will increase at the same injection volume, which will lead to the injection pressure increase according to Darcy's law. Thus, the injection pressure can be used to measure the plugging effect of PMs (Liu et al., 2017). After the PMs' injection, the more the injection pressure increases, the better the plugging effect. For the thickness of the water-absorbing layer, after the high permeability channel is plugged, the subsequent injected water must bypass the plugged area to continue flowing, and that means the thickness of the water-absorbing layer will increase (Jia et al., 2018). So, it also can be used as one of the parameters to measure the plugging effect of PMs. After the PMs' injection, the more the thickness of the water-absorbing layer increases, the better the plugging effect. The thickness of water-absorbing can be measured using the isotopic tracer technique. Its main principle is that radioisotopes flow into the reservoir with injected water and are adsorbed on the surface of the water-absorbing layer. Then, the gamma instrument is used to measure the radiation intensity of the isotope. According to the measurement results, the thickness of the water absorption layer can be calculated by the software of the isotope tracing test (Liu et al., 2020).

**Figure 12:**
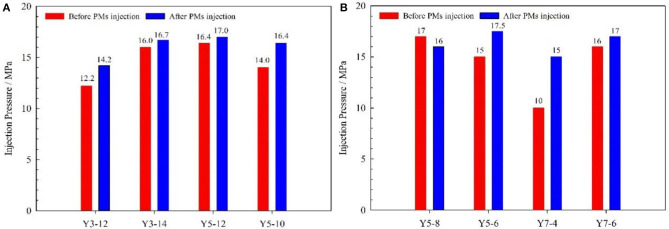
Injection pressure before and after PMs injection. **(A)** The calibration diameter is 800 nm; **(B)** The calibration diameter is 300 nm.

**Figure 13:**
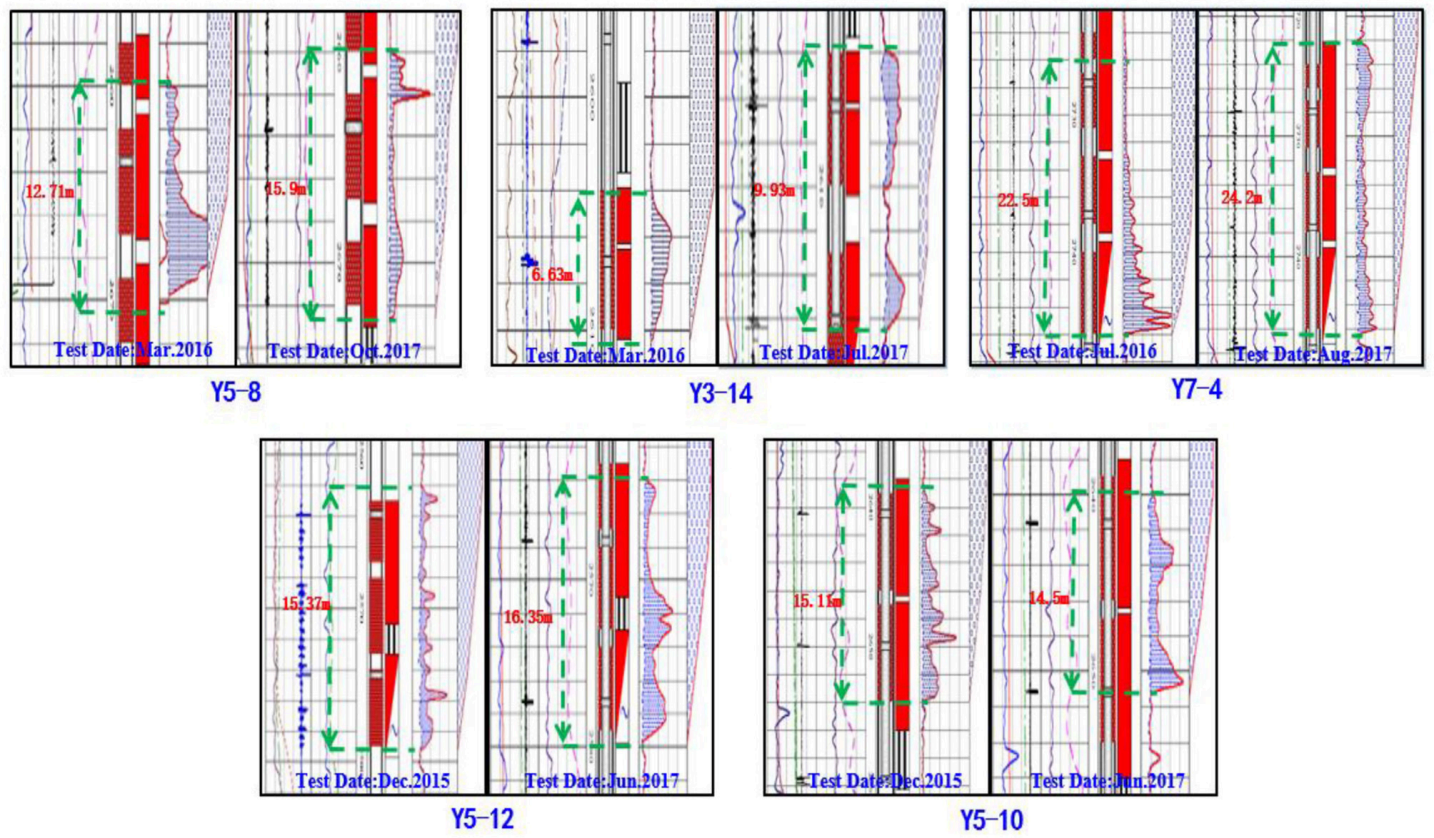
Results of the water injection profile test before and after the PMs injection.

For the injection well in the pilot test area, the average daily injection rate of a single well was 21.2 t/d after PMs' injection. Comparing the injection rate before and after PMs' injection, it showed only a little change. The injection pressure significantly increased for most injection wells, as shown in Figure 12. The average injection pressure of a single well was 16.2 MPa, and increased by 1.6 MPa comparing the injection pressure before PMs' injection. The results of the water injection profile test before and after PMs' injection are shown in Figure 13, and the detailed data of the water injection profile test before and after PMs' injection are shown in Table 5. Comparing the results of the water injection profile test before and after PMs' injection, the characteristic of non-uniform water-absorbing was significantly meliorated, and the average thickness of the water-absorbing layer increased from 14.46 to 16.17 m.

**Table 5:** Data of the water injection profile test before and after the PMs injection.

**Well**	**Particle size (nm)**	**Before PMs injection**		**After PMs injection**	
		**Test time**	**TWAL^a^(m)**	**Test time**	**TWAL^a^(m)**
Y3-14	800	2016.03	6.63	2017.07	9.91
Y5-10		2015.12	15.11	2017.06	14.50
Y5-12		2015.12	15.37	2017.06	16.35
Y5-8	300	2016.03	12.71	2017.10	15.90
Y7-4		2016.07	22.50	2017.08	24.20
	Average value		14.46		16.17

a *Thickness of water-absorbing layer*.

For the four injection wells using the PMs with a calibration diameter of 800 nm, the injection pressure was increased after PMs' injection, and its value increased by 2.0, 0.7, 0.6, and 2.4 MPa, respectively, as shown in Figure 12A. The average injection pressure of a single well increased by 1.4 MPa, and the average thickness of the water-absorbing layer increased by 1.22 m. For the four injection wells using the PMs with the calibration diameter as 300 nm, the injection pressure of 3 injection wells increased and that of one injection well decreased after PMs injection, and its value increased by 2.5 MPa, 5.0 MPa, 1.0 MPa and decreased by 1.0 MPa as shown in Figure 12B. The average injection pressure of a single well-increased by 1.9 MPa, and the average thickness of the water-absorbing layer increased by 2.81 m.

For the injection wells after the PMs' injection, the injection pressure significantly increased and the thickness of the water-absorbing layer significantly increased. These phenomena indicate that the PMs effectively plug the high-permeability channel in the reservoir, and significantly meliorate the characteristic of non-uniform water-absorbing. In the pilot test, the water-flooding conditions improved significantly after PMs' injection, and the application of PMs for in-depth profile control has a significant effect. The dynamic data of the injection wells shows the increased margin of average injection pressure and average water absorbing layer thickness for the injection wells using the PMs with a calibration diameter of 300 nm is larger than the injection wells using the PMs with a calibration diameter of 800 nm. It indicated that, for the injection wells in the pilot test area, the PMs with a calibration diameter of 300 nm are better than the PMs with a calibration diameter of 800 nm.

#### Production Well Performance Evaluation

The application of PMs for in-depth profile control is used mainly to improve the volumetric sweep efficiency of water-flooding (Raffa et al., 2016). When the high-permeability channel is plugged by PMs, the water will flow around, and then the swept volume of water-flooding will increase. Therefore, the oil layers that have not been swept before will be swept. In turn, oil production will increase. However, not all the oil production of production wells will increase after PMs' injection. It depends on many factors. The high-permeability channels in the reservoir can be plugged by the PMs, but are not plugged forever. The PMs will gradually cease to be in effect with water continually flooding. That is to say, there is a validity period for PMs plugging high-permeability channels. So, the validity period can be used as one of the parameters to evaluate the plugging ability of PMs. Other evaluation parameters include the success ratio, oil increment, and non-uniformity coefficient of liquid production and water cut.

**Figure 14:**
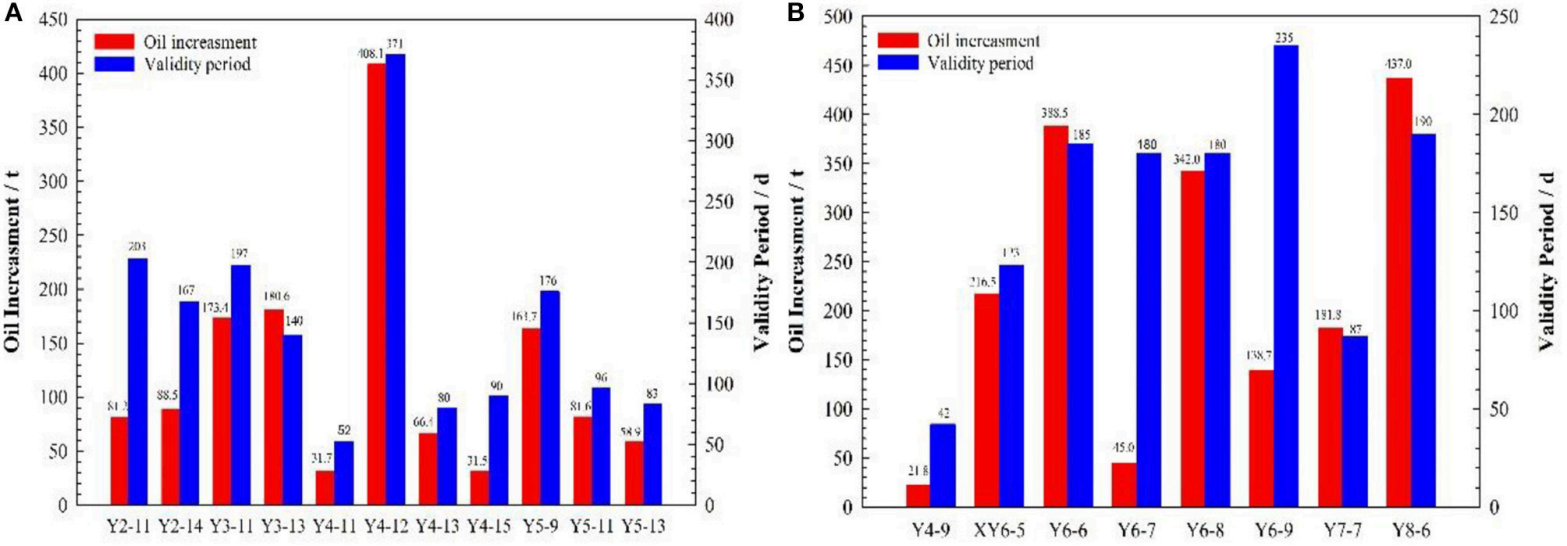
Oil production increment and validity period for production wells. **(A)** The calibration diameter is 800 nm; **(B)** The calibration diameter is 300 nm.

For the production wells in the pilot test area, after using the PMs for in-depth profile control, the oil production of 19 wells out of the 42 wells increased, and the success ratio of in-depth profile control was 45.3%. The average daily liquid production of a single well-increased from 5.8 to 6.7 t/d, and the average water cut of a single well-decreased from 60.6 to 50.2%. The non-uniformity coefficient of liquid production decreased from 0.66 to 0.59, and the non-uniformity coefficient of water cut decreased from 0.45 to 0.4. The validity period and total oil production increment of single wells are shown in Figure 14. For all the response production wells, the maximum and minimum validity period was 371 d and 42 d, respectively, and the average validity period was 151 d. The total oil production increment of production wells was 3,136.9 t, of which the maximum oil production increment of single wells was 437 t, and the minimum oil production increment of single wells was 21.8 t. After the PMs were injected into the pilot test, the production conditions of production wells improved, and the oil production increased significantly.

For the 23 production wells using the PMs with a calibration diameter of 800 nm, the oil production of 11 wells out of the 23 wells increased, and the success ratio of in-depth profile control was 47.8%. The average daily liquid production of single wells increased from 5.2 to 6.2 t/d, and the average water cut of single wells decreased from 58.3 to 55.6%. The non-uniformity coefficient of liquid production decreased from 0.63 to 0.56, and the non-uniformity coefficient of water cut decreased from 0.51 to 0.45. The validity period and total oil production increment of single wells are shown in Figure 14A. For all the response production wells, the maximum and minimum validity period was 371 d and 52 d, respectively, and the average validity period was 150 d. The total oil production increment of production wells was 1,365.6 t, of which the maximum oil production increment of single wells was 408.1 t, and the minimum oil production increment of single wells was 31.5 t.

For the 21 production wells using the PMs with a calibration diameter of 300 nm, the oil production of eight wells out of the 21 wells increased, and the success ratio of in-depth profile control was 38.1%. The average daily liquid production of single wells increased from 6.2 to 7.3 t/d, and the average water cut of single wells decreased from 61.6 to 47.8%. The non-uniformity coefficient of liquid production decreased from 0.72 to 0.61, and the non-uniformity coefficient of water cut decreased from 0.42 to 0.33. The validity period and total oil production increment of single wells are shown in Figure 14B. For all the response production wells, the maximum and minimum validity period was 235 d and 42 d, respectively, and the average validity period was 153 d. The total oil production increment of production wells was 1,771.3 t, of which the maximum oil production increment of single wells was 437 t, and the minimum oil production increment of single well was 21.8 t.

For the production well after the PMs injection, the average daily liquid production of single wells significantly increased, and the water cut, the non-uniformity coefficient of liquid production, and the non-uniformity coefficient of water cut significantly decreased. This phenomenon shows that the efficiency of water-flooding has been significantly improved. Combined with other evaluation parameters, such as the success ratio, validity period, and oil increment, the evaluation results indicate that the application of PMs for in-depth profile control in the pilot test area was very successful. Comparing the dynamic data of production wells using the PMs with a calibration diameter of 800 nm and with a calibration diameter of 300 nm, after PMs' injection, the performance evaluation parameters of the wells using the PMs with a calibration diameter of 300 nm are better than the wells using the PMs with a calibration diameter of 800 nm. The total oil increment and average validity period for the production wells using the PMs with a calibration diameter of 300 nm are larger than the production wells using the PMs with a calibration diameter of 800 nm, but the success ratio of in-depth profile control is the opposite. These evaluation parameters indicate that, for the production wells in the pilot test area, the PMs with a calibration diameter of 300 nm are better than the PMs with a calibration diameter of 800 nm.

After the application of PMs in the pilot test area for in-depth profile control, the production conditions of injection wells and production wells improved significantly. For the production wells, the water cut decreased, and the oil production increased. For the injection wells, the characteristic of non-uniform water-absorbing profile was improved. For the pilot test area, the efficiency of water-flooding improved significantly. These characteristics indicate that the PMs can be used for in-depth profile control in ultralow permeability oil reservoirs. Comparing the dynamic data of production wells and injection wells using the PMs with a calibration diameter of 800 nm and with a calibration diameter of 300 nm, it indicates that, for the pilot test area, PMs with a calibration diameter of 300 nm are more suitable than the PMs with a calibration diameter of 800 nm.

## Conclusion

This paper reports on a field application case of nanoscale PMs for in-depth profile control in an ultralow permeability oil reservoir. In the paper, the PMs with calibration diameters of 300 and 800 nm were selected, and evaluation experiments were done to ensure their properties. Then, the performance of PMs in the pilot test area was evaluated, and the more suitable PMs size was determined for the pilot test area. The major conclusions that could be drawn from this study are as follows:

(1) Laboratory experiment results indicate that the PMs have a good capacity of swelling and plugging. More specifically, for the PMs with a calibration diameter of 300 nm, the final equilibrium swelling ratio is 56.2 nm·nm^−1^, and the maximum resistance coefficient of the blocking rate after swelling are 3.7 and 70.31%, respectively. For the PMs with a calibration diameter of 800 nm, the final equilibrium swelling ratio is 49.4 nm·nm^−1^, and the maximum resistance coefficient of the blocking rate after swelling are 3.5 and 71.42%, respectively.
(2) Comparing the dynamic data of well groups using the PMs with a calibration diameter of 800 nm and with a calibration diameter of 300 nm, it indicates that, for the pilot test area, PMs with a calibration diameter of 300 nm are more suitable than the PMs with a calibration diameter of 800 nm.
(3) The performance evaluation results indicate that the PMs can be used for in-depth profile control in ultralow permeability oil reservoirs. Specifically, after the application of PMs in the pilot test area for in-depth profile control, for the production wells, the water cut decreased, and the oil production increased. For the injection wells, the characteristic of non-uniform water-absorbing profile improved. For the pilot test area, the efficiency of water-flooding improved significantly.

## Data Availability Statement

The raw data supporting the conclusions of this article will be made available by the authors, without undue reservations.

## Author Contributions

GH participated in the design of the manuscript and drafted the manuscript. GH, WZ, and YJ came up with ideas for the manuscript. XY and JZ carried out experiments. XY, GH, and TL analyzed experimental results. JZ and GH evaluated the performance of PMs in the pilot test area. TL and JH revised the manuscript. All authors contributed to the article and approved the submitted version.

## Conflict of Interest

YJ was employed by the Oil & Gas Technology Research Institute of Changqing Oilfield Company and the JZ was employed by the Beijing Jinshi Liyuan Science Co., Ltd. The remaining authors declare that the research was conducted in the absence of any commercial or financial relationships that could be construed as a potential conflict of interest.
